# Validation of two *Amanita* species from eastern North America: *A.rhacopus* sp. nov. and *A.variicolor* sp. nov.

**DOI:** 10.3897/mycokeys.38.27041

**Published:** 2018-08-14

**Authors:** Herman Lambert, Guy Fortin, Roland Labbé, Jacqueline Labrecque, Jean A. Bérubé, Jacques Landry, Evgeny Ilyukhin, Simona Margaritescu, Jean-Marc Moncalvo, Yves Lamoureux

**Affiliations:** 1 Mycoquébec, 1313, rue Louis-Francoeur, Québec, Canada G1Y 1N7 Mycoquébec Québec Canada; 2 Ressources naturelles Canada, Service canadien des forêts, Centre de foresterie des Laurentides, 1005, rue du P.E.P.S., C.P. 10380, Québec, Canada G1V 4C7 Centre de foresterie des Laurentides Québec Canada; 3 Department of Natural History, Royal Ontario Museum, Toronto, Canada M5S 2C6 Department of Natural History, Royal Ontario Museum Toronto Canada; 4 Department of Ecology and Evolutionary Biology, University of Toronto, Toronto, Canada M5S 3B2 University of Toronto Toronto Canada

**Keywords:** *
Amanitaceae
*, Agaricales, taxonomy

## Abstract

Members of the mushroom genus *Amanita* usually can easily be identified to the genus in the field, however, species circumscription and identification are often problematic. Several names have been misapplied and cryptic species exist. Here, we formally describe and validate two new species of Amanitasect.Vaginatae from eastern North America that were recognised under the umbrella European names *A.ceciliae* by past authors: *Amanitarhacopus***sp. nov.** and *Amanitavariicolor***sp. nov.**

## Introduction

The genus *Amanita* is one of the best-known genera within the Agaricales. The genus contains edible and poisonous mushrooms, is distributed worldwide and is entirely or mostly ectomycorrhizal depending on which side one stands in its currently debated circumscription (Redhead et al. 2016, Tulloss et al. 2016, Hawksworth 2016). Many *Amanita* species are common, quite robust and readily recognisable in the field. However, cryptic species exist and several European names have been misapplied to taxa from other continents. In eastern North America, more than 75 species are documented and well characterised although several taxa are still not validly published (Lamoureux 2006, Tulloss 2017).

Following an extensive survey conducted by one of us in the province of Québec, Canada, between 1985 and 2005, it was concluded that the region hosts at least 52 taxa of *Amanita* (Lamoureux 2006), which is about twice the 27 species reported by Pomerleau in 1980 for the same region. Amongst these taxa, 11 appear new to science, not corresponding to any known American or European species. Although these species were provisionally named (Lamoureux 2006), the names are not valid since the descriptions of the species lacked a diagnosis and a reference to a holotype.

Two of the new species proposed by Lamoureux (2006) corresponded to what Pomerleau (1980) originally called *A.ceciliae* (Berk. & Broome) Bas or its later synonym *A.inaurata* Secr. ex Gillet. *A.ceciliae* is a European taxon first described in 1854 and this name was later used worldwide to label species with similar appearance. These two new species were described as medium size *Amanita* with cap striated at the margin, a cylindrical stipe without annulus and a friable universal veil, greyish with age with orange tint or not, often leaving some remnants on the cap and at the stipe base. *A.rhacopus* Y. Lamoureux nom. prov. is brown to dark greyish-brown whereas *A.variicolor* Y. Lamoureux nom. prov. has variable intensity of pileus colour but differs mainly by its orange tint especially at the stipe base. Both are in conifer forest mixed with *Betula*. The concept of these two *Amanita* species became well accepted in eastern North America, although *A.ceciliae* ss. auct. amer. is still used by many for both species to avoid the use of *provisorum nomen* (see Tulloss 2017). Recently, DNA sequences of several eastern North America and Québec collections of *A.rhacopus* and *A.variicolor* were released in Genbank (R. Tulloss, unpublished work). The present work aims at validating these two names by designating holotypes and providing diagnoses, detailed descriptions as well as ITS-rDNA barcode sequences (Schoch et al. 2012).

## Materials and methods

### Specimens examined

Type specimens are deposited in the Cercle des mycologues de Montréal Fungarium, (CMMF). Additional specimens are in the Royal Ontario Museum Fungarium (TRTC), in CMMF or in the private collections of H. Lambert (HL), R. Lebeuf (HRL) or R. Labbé (RLA).

### Morphological examination

Collections examined in this study were photographed in the field and macromorphological features were derived from both field notes and pictures. Microscopic studies were performed in saline solution on fresh material or in 3% ammoniac on exsiccata. Melzer’s reagent was used for amyloidity, Cotton Blue for cyanophily and Congo Red for tissue staining. Dimensions of microscopic elements are given as: [a/b/c] (min) D1-D9 (max) where a, b and c represent the number of elements measured, the number of specimens and collections from which the elements were studied, respectively; min and max, the extreme values of the distribution; D1 and D9, the first and ninth decile. Q denotes the length/width ratio of a basidiospore in side view, Qm refers to the arithmetical mean. All microscopic elements were obtained using a Leitz Ortholux II or an Olympus CH-2 microscope equipped with digital camera and were measured from pictures using Piximetre software v.5.6 (Alain Henriot, France).

## DNA sequencing and analyses

ITS-rDNA barcode sequences were obtained following Dentinger et al. (2010). DNA was extracted from dried herbarium specimens (Québec collections) or from fresh tissue blotted on FTA cards (Ontario collections). BLAST searches (Altschul et al. 1990) were conducted in Genbank and in UNITE (Kõljalg et al. 2005, Abarenkov et al. 2010) to compare the new sequences with those available in these databases.

## Results

### DNA sequence analyses

The sequences we obtained from seven *A.rhacopus* collections were identical to each other, whereas those obtained from three specimens of *A.variicolor* differed at two nucleotide positions. The ITS sequences of the holotypes were subjected to BLAST searches against the GenBank and UNITE databases. The holotype sequence of *A.rhacopus* CMMF002171 retrieved 20 sequences with 100% identity deposited as *Amanita* sp. ‘rhacopus’ by Tulloss et al. of specimens from Connecticut (KY435399, KP224337), Pennsylvania (KX061516, KX270322, KX270321, KX270320, KX270319), Maine (KP224336, KU186825, KP221312, KP662537, KP221311), New York (KP224339), New Jersey (KP224333, KP224331, KP224329), West Virginia (KP224332, KP224330), Texas (KP224334), Tennessee (KP224335) and Québec (KP224338). Then follow several species of Amanitasect.Vaginatae having sequence similarities of 96% or less, including unnamed species.

The ITS sequence of the holotype of *A.variicolor* CMMM003787 has highest similarities with two sequences deposited in Genbank as *Amanita* sp. ‘*variicolor*’, one by Tulloss et al. (KP711844, specimen CMMF003463 from Québec; unpublished) and one by Bérubé et al. (KJ638268, specimen HL0846, also from Québec; unpublished), showing, respectively, zero to four mismatches out of 509 aligned nucleotides. Other highly similar sequences were from collections labelled *Amanita* sp., one from Arizona (MG518639, T.A. Clements, unpublished, which differs at six sites) and one from North Carolina (AY456335, Edwards et al. 2004, which differs at nine sites). Then follow several species of sect. Vaginatae with 93% similarity or less.

ITS sequences from *A.rhacopus* and *A.variicolor* differ by 8%. They were clearly distinct from a bona fide European sequence of *A.ceciliae* (13% difference from UNITE sequence UDB002316 | RK639).

### Taxonomy

#### Amanita rhacopus

Taxon classificationFungiAgaricalesAmanitaceae

Y. Lamoureux sp. nov.

827343

Fig. 1

A. inaurata ss. Pomerl. p. p.; A.ceciliae ss. auct. amer. p. p.
non Amanitainaurata Secr. ex Gillet, Hyménomycètes (Alençon): 41 (1874) [1878]
non Agaricusceciliae Berk. & Broome, Ann. Mag. nat. Hist., Ser. 2 13: 396 (1854)

##### Diagnosis.

*Amanitarhacopus* differs from other species of AmanitasectionVaginatae by its brown to dark grey-brown pileus, stipe white at times covered with grey chevrons, universal veil grey leaving small to large flakes on pileus and annulus-like remnants at the stipe base, found in stands of conifers (*Abies*, *Picea*, *Pinus*, *Tsuga*) mixed with *Betula*.

##### Holotype.

CANADA, Québec: Mont Orford, in mountain, close to a stand of *Betulapapyrifera* in a *Abiesbalsamea* and *Tsugacanadensis* forest, 45°18'43" North, 72°14'24" West, 11 July 1994, CMMF002171, ITS Genbank accession number MG734660.

**Description.** Pileus 40-80 mm wide, ovoid to rounded conic slightly umbonate to applanate, smooth, brown to greyish-brown, with time darker in the centre and over inner ends of marginal striations, often with grey velar flakes, margin striated. Lamellae free, crowded, greyish near the pileus margin or completely greyish with age, lamellulae numerous, truncated, of very diverse lengths, unevenly distributed, edges finely powdered, white to whitish. Stipe 70–120 × 7–13 mm, cylindrical (not bulbous), flocculose and white first, then smooth to appressed fibrillose, whitish to greyish, at times with chevron-forming greyish fibrils, without annulus, with grey annulus-like remnants at the base. Universal veil friable, grey, leaving flakes on the pileus and annulus-like remnants at the stipe base. Partial veil absent. Context whitish, unchanging when cut or bruised, odour and taste not distinctive.

Basidiospores [474/11/10] (8.4) 9.5–11.7 (14.5) × (7.9) 9.0–11.1 (13.7) µm, Q= 1.0–1.1(1.2), Qm=1.05, globose to subglobose, smooth, monoguttulate, hyaline, inamyloid and cyanophilous. Basidia (50) 60–75 × 14–16 (18) µm, clavate, usually 4-spored with 4–6 μm long sterigmata, occasionally 2-spored with 5–10 µm long sterigmata, clampless. Subhymenium composed of irregular globose to subglobose 9–18 × 6–9 µm cells. Lamellar trama bilateral consisting of cylindro-clavate, clavate, fusiform to subfusiform, abundantly inflated cells 40–65 × 7–18 µm, mixed with thin-walled, hyaline, 3–6 μm wide filamentous hyphae and of rare 3–6 µm wide, sinuous vascular hyphae. Volva remnants composed of short 3–7 µm wide filamentous ramified hyphae, numerous 25–50 µm wide terminal globose cells (few subglobose), rare to absent vascular hyphae. Pileipellis composed of 4–12 µm wide interwoven gelatinised brown filamentous hyphae mixed with an equal amount of 45–100 × 8–22 µm inflated cylindrical cells, often in chains and some 4–7.5 µm wide vascular hyphae. Pileus context composed of 4–12 µm wide filamentous sometimes partially inflated hyphae and 70–170 × 15–30 µm cylindrical to clavate inflated cells, often in chains with cells of the same diam. and some 4–8.5 µm wide vascular hyphae, ramified and distributed in all parts of the context. Stipitipellis composed of 40–180 (270) × (16) 20–30 (35) µm clavate terminal cells with grey pigment encrusted wall, originating from undifferentiated 4–6 µm wide hyphae. Stipe context composed mainly of 120–360 × 20–50 µm cylindrical cells in chains with ramified 3–5 µm wide filamentous hyphae and 4–7 µm (apex) and 5–23 µm (centre) wide vascular hyphae. Clamps absent.

**Figure 1.**
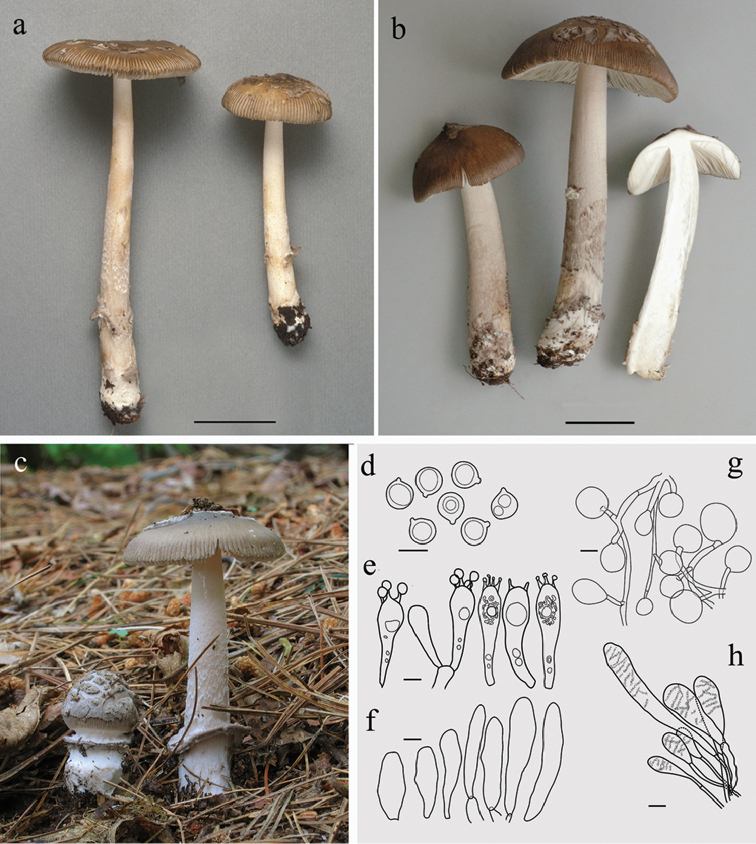
*Amanitarhacopus*. **a–c** Basidiomes **a** CMMF002171(holotype), photograph by Yves Lamoureux **b** CMMF009640, photograph by Jacqueline Labrecque **c** HL016, photograph by Herman Lambert **d–h** Drawings of typical microscopic structures by Guy Fortin **d** Basidiospores **e** Basidia **f** Acrophysalides **g** Universal veil. h. Caulocystides. Scale bar: 3 cm (**a, b**), 10 µm (**d, e**), 20 µm (**f–h**).

##### Ecology and distribution.

Solitary or scattered in stands of conifers (*Abies*, *Picea*, *Pinus*) mixed with *Betula*, on mesic to sub-mesic soil, never seen in plantations, from July to September in Québec and, according to sequences in Genbank, in all eastern North America down to Tennessee and Texas.

##### Etymology.

The epithet *rhacopus* refers to the Greek ῤάκος, meaning piece of cloth and πούς, meaning foot.

##### Specimens examined.

Canada, Québec: Québec, Boisé de l’aéroport, R. Labbé (RLA30465), 4 August 2007. Québec, Base de plein air La Découverte, H. Lambert (HL0787), 10 July 2010. Sainte-Catherine-de-la-Jacques-Cartier, Station touristique Duchesnay (sentier 51), H. Lambert (HL002), 7 July 2012. Lac-Beauport, H.Lambert (HL016), 12 July 2008. Québec, Château-Bigot, H. Lambert (HL049), 21 September 2014 (Genbank accession number MG734661). Québec, Base de plein air La Découverte, H. Lambert (HL022), 7 July 2013. Lac-Beauport, Lac Neigette nord, J. Labrecque (CMMF009600), 24 July 2007. Lac-Beauport, Chemin de la Chapelle, J. Labrecque (CMMF008929), 11 August 2006. Québec, Boisé de l’aéroport, R. Labbé (RLA30063), 15 July 2006. Lac-Beauport, Chemin de la Chapelle, J. Labrecque (CMMF009640), 29 July 2007 (Genbank accession number MG734662). Saint-Raymond, lac Sept-Iles, R. Lebeuf (HRL1876), 27 September 2014 (Genbank accession number MG734658). Québec, Château-Bigot, H. Lambert (HL048), 21 September 2014 (Genbank accession number MG734663). Grondines, Highway 40, Renée Lebeuf (HRL0804), 19 August 2011 (Genbank accession number MG734664). Ontario: Algonquin Provincial Park, M. Didukh and B. Dentinger (TRTC156853), 29 September 2007 (Genbank accession number MG734659).

#### Amanita variicolor

Taxon classificationFungiAgaricalesAmanitaceae

Y. Lamoureux sp. nov.

827344

Fig. 2

A. inaurata ss. Pomerl. p. p.; A.ceciliae ss. auct. amer. p. p.
non Amanitainaurata Secr. ex Gillet, Hyménomycètes (Alençon): 41 (1874) [1878]
non Agaricusceciliae Berk. & Broome, Ann. Mag. nat. Hist., Ser. 2 13: 396 (1854)

##### Diagnosis.

*Amanitavariicolor* differs from other species of Amanitasec.Vaginatae by its versicolour (straw-yellow, orange-brown to blackish brown) pileus, stipe white then covered with brown olive to orange chevron-forming fibrils, stipe base dark orange to rusty, universal veil grey to orange-grey leaving small to large flakes on the pileus and one or two strips at the stipe base, found mainly with *Abies* and *Betula*.

##### Holotype.

CANADA, Québec: Rawdon, in a mixed forest of *Abiesbalsamea* and *Betulapapyrifera*, on moist soil close to a bog, 19 August 2003, CMMF003787, ITS Genbank accession number MG734656.

##### Etymology.

The epithet *variicolor* refers to the very variable colour of the pileus.

##### Description.

Pileus 40–100 mm wide, ovoid to rounded conic at first, then plane with an umbo, smooth, olive yellow, straw yellow, bronze, olive brownish to brown-black and then tinged with olive or orange yellow towards the margin, at times darker in the centre and over inner ends of marginal striations, often with small to large grey or orange grey velar flakes, margin striated. Lamellae free, subcrowded, whitish, greyish to salmon tints. Stipe100–200 × 8–17 mm, cylindrical (not bulbous or barely), flocculose and white at first, typically covered all over by chevron-forming rusty-orange fibrils on a whitish background when mature, without annulus, base always rusty orange with one or two greyish-orange velar strips. Universal veil friable, grey to orange grey, often leaving small to large flakes on the pileus and one or two strips at the stipe base. Partial veil absent. Context white, unchanging when cut or bruised, odour not distinctive, taste not recorded.

Basidiospores: [180/3/3] (8.2) 9.8–11.5 (13.3) × (7.1) 8.8–10.7 (12.2) µm, Q= 1.0–1.2 (1.3), Qm= 1.09, globose to subglobose, smooth, monoguttulate, hyaline, inamyloid and cyanophilous. Basidia 48–65 × 14–19 µm, clavate, 4-spored with sterigmata up to 8.5 μm long. Subhymenium composed of irregular globose to subglobose 11–20 (25) × (6) 10–15 µm cells. Lamellar trama bilateral consisting of cylindro-clavate, clavate, fusiform to subfusiform, abundantly inflated 28–40 (55) × 13–20 µm cells mixed with thin-walled, hyaline, 2–6 μm wide filamentous hyphae and rare vascular hyphae. Volva remnants composed of 4.5–7.5 µm wide filamentous hyphae terminated by 25–60 µm wide subglobose to globose and inflated cells often in chains and rare to absent vascular hyphae. Pileipellis composed of an upper layer of 2.5–6 µm wide radially orientated gelatinised hyphae and a subpellis of mainly filamentous 4–12 µm wide hyphae mixed with cylindrical to fusiform inflated 50–100 × 13–24 µm cells often in chains and some 7–10 µm wide vascular hyphae. Pileus context composed of equal amounts of (4) 5–12 µm wide filamentous hyphae, sometimes inflated, more or less ramified and cylindrical, subfusiform to fusiform 40–110 × 10–33 µm inflated cells often in chains, with some 7–10 (12) µm wide vascular hyphae, sometimes inflated, rarely ramified. Stipitipellis composed of 3–5 (6) µm wide filamentous hyphae terminated by clavate 50–90 (120) × (12) 16–23 µm cells containing reddish-brown pigments. Stipe context composed mainly of cylindrical 150–350 × 20–35 µm cells in chains, 4–13 µm wide filamentous hyphae and some 7–10 µm wide vascular hyphae. Clamps absent.

**Figure 2.**
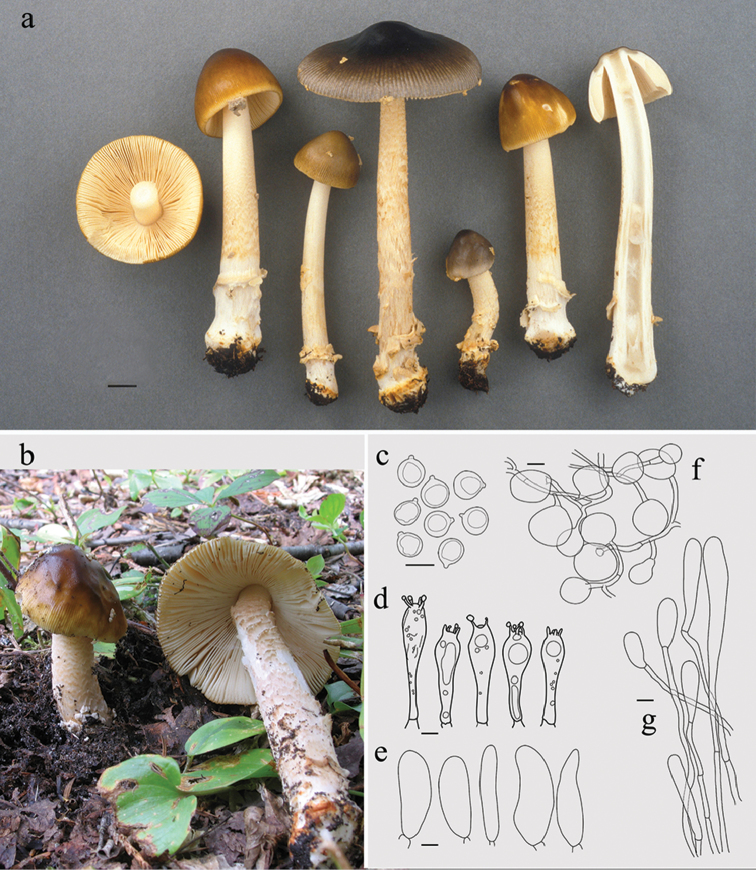
*Amanitavariicolor*. **a, b** Basidiomes **a** CMMF003787 (holotype), photograph by Yves Lamoureux **b** HL0257, photograph by Herman Lambert **c–g** Drawings of typical microscopic structures by Guy Fortin **c** Basidiospores **d** Basidia **e** Acrophysalides. f. universal veil **g** Caulocystides. Scale bar: 1 cm (**a**), 10 µm (**c, d**), 20 µm (**e–g**).

##### Ecology and distribution.

Solitary, sometimes scattered to gregarious, in stands of conifers (*Abies*, *Picea*, *Pinus, Tsuga)* mixed with *Betula*, on sub-hydric to mesic soil, never seen in plantations, from July to September in Québec and Ontario. Possibly also present further south (see Discussion).

##### Specimens examined.

Canada, Québec: Québec, Base de plein air La Découverte, H. Lambert (HL0846), 15 August 2010. Lac-Beauport, Chemin de la Chapelle, H. Lambert (HL0852), 22 August 2010. Sacré-Sœur-sur-le Fjord, Rivière Sainte-Marguerite, fosses 3, 4, 5, H. Lambert (HL0257), 17 August 2008 (Genbank accession number MG734657). Sacré-Sœur-sur-le Fjord, Parc Saguenay, H. Lambert (HL051), 2015. Ontario : Algonquin Provincial Park, M. Didukh and B. Dentinger (TRTC156902), 1 October 2007 (Genbank accession number MG734655).

## Discussion

*Amanitarhacopus* and *A.variicolor* belong to subgenus AmanitasectionVaginatae (Fr.) Quél. due to the absence of a basal bulb and a partial veil, inamyloid basidiospores and absence of clamp connections at the base of basidia (Bas 1969, Yang 1997). Tulloss (2017) lists 296 names in this section, of which 97 correspond to accepted species, 132 are cryptonyms and 57 are provisory names. Many ITS sequences from members of sect. Vaginatae have been deposited in public databases, however, most are unidentified, misidentified or refer to provisory names. In addition, ITS variation within the section is very high and comprises numerous insertion/deletion events that preclude unambiguous sequence alignment in many positions. For these reasons, we have refrained to place *A.rhacopus* and *A.variicolor* in an ITS phylogeny.

In general, *A.variicolor* is larger than *A.rhacopus*. The pileus of *A.rhacopus* is greyish-brown becoming darker with age, never with an orange tint as found on *A.variicolor*. The lamellae of *A.rhacopus* become greyish with time, whereas the lamellae of *A.variicolor* become greyish-salmon. The universal veil texture of *A.rhacopus* is stronger than in *A.variicolor*, leaving a pseudo-annulus at the stipe base, which is more distinct on young basidiomes. In contrast, the universal veil of *A.variicolor* leaves light orange strips on the stipe base. The basal extremity of the stipe is always rusty orange in *A.variicolor*, a characteristic which is never seen in *A.rhacopus*. Both species developed chevron-like motifs on the stipe surface and are growing under conifers and *Betula* mixed forest. *A.variicolor* appears to be less frequent than *A.rhacopus*. They were never observed in pure plantations, only in native forest. *A.variicolor* is growing on rich mossy soil, *A.rhacopus* on dry soil often in spine conifer litter.

Although nucleotide sequence variation alone cannot be used for the circumscription of fungal species, empirical observations as well as a comprehensive study by Nilsson et al. (2008) indicated that ITS intraspecific variation in the Basidiomycetes is typically in the 0-3% range. Here, we found that ITS sequences amongst 27 collections of *A.rhacopus*, from Québec to Texas, were identical and differ from at least 4% from other *Amanita* sequences that have been deposited in public databases so far. This fact, along with its morphological uniqueness, strongly supports the recognition of *A.rhacopus* as a new and widespread species in eastern and southern North America.

While *A.rhacopus* shows remarkable ITS sequence homogeneity from Québec to Texas, *A.variicolor* sequences vary by 0.78% amongst collections from Québec and Ontario and differ by 1.76% from two sequences deposited in Genbank as *Amanita* sp., one from North Carolina (AY456335) and one from Arizona (MG518639). The former sequence comes from a fruiting body collected in a loblolly pine forest, whereas the latter was retrieved from a soil sample under ponderosa pine, douglas fir and gambel oak. Whether or not these two sequences belong to *A.variicolor* needs further investigation. If this is the case, then the distribution range of this species would also encompass areas in the eastern and southern U.S.A.

## Supplementary Material

XML Treatment for Amanita rhacopus

XML Treatment for Amanita variicolor
